# Glucose Supply Induces PsMYB2-Mediated Anthocyanin Accumulation in *Paeonia suffruticosa* ‘Tai Yang’ Cut Flower

**DOI:** 10.3389/fpls.2022.874526

**Published:** 2022-06-14

**Authors:** Lili Zhang, Li Yan, Chao Zhang, Xin Kong, Yiqing Zheng, Li Dong

**Affiliations:** ^1^Beijing Key Laboratory of Ornamental Plants Germplasm Innovation and Molecular Breeding, National Engineering Research Center for Floriculture and College of Landscape Architecture, Beijing Forestry University, Beijing, China; ^2^Ningxia State Farm, Yinchuan, China; ^3^Zhejiang Provincial Key Laboratory of Germplasm Innovation and Utilization for Garden Plants, School of Landscape Architecture, Zhejiang Agriculture and Forestry University, Hangzhou, China

**Keywords:** cut flower, anthocyanin biosynthesis, tree peony, PsMYB2, glucose signal

## Abstract

Tree peony (*Paeonia suffruticosa*) is a well-known Chinese ornamental plant with showy flower color. However, the color fading problem during vase time seriously blocks its development in the cut flower market. In this study, we found that exogenous glucose supply improved the color quality of *P. suffruticosa* ‘Tai Yang’ cut flowers with increased total soluble sugar and anthocyanin contents of petals. Besides, the promotion effect of glucose was better than the osmotic control of 3-*O*-methylglucose (3OMG) treatment and the glucose analog mannose treatment. The structural genes, including *PsF3H*, *PsF3′H*, *PsDFR*, *PsAOMT*, and *PsUF5GT*, were remarkably upregulated under glucose treatment. Meanwhile, the regulatory genes, including *PsbHLH1*, *PsbHLH3*, *PsMYB2*, *PsWD40-1*, and *PsWD40-2*, also showed a strong response to glucose treatment. Among these five regulatory genes, *PsMYB2* showed less response to 3OMG treatment but was highly expressed under glucose and mannose treatments, indicating that *PsMYB2* may have an important role in the glucose signal pathway. Ectopic overexpression of *PsMYB2* in *Nicotiana tabacum* resulted in a strong pigmentation in petals and stamens of tobacco flowers accompanied with multiple anthocyanin biosynthetic genes upregulated. More importantly, the overexpression of *PsMYB2* enhanced the ability of glucose-induced anthocyanin accumulation in *Arabidopsis thaliana* seedlings since *PsMYB2*-overexpressing *Arabidopsis* showed higher expression levels of *AtPAL1*, *AtCHS*, *AtF3H*, *AtF3′H*, *AtDFR*, and *AtLDOX* than those of wild type under glucose treatment. In summary, we suggested that glucose supply promoted petal coloration of *P. suffruticosa* ‘Tai Yang’ cut flower through the signal pathway, and *PsMYB2* was a key component in this process. Our research made a further understanding of the mechanism that glucose-induced anthocyanin biosynthesis of *P. suffruticosa* cut flowers during postharvest development, laying a foundation for color retention technology development of cut flowers.

## Introduction

In China, due to the various flower colors and long cultivation history, tree peony possesses the reputation of “the King of Flowers” and becomes a competitive candidate for “Chinese National Flower” (Suo et al., 2008). However, a fast decrease in the ornamental quality along with color fading that always appears in the cut flowers during vase time greatly impairs its commercial value as cut flower. Therefore, color quality declining of tree peony during postharvest development is urgently needed to be solved.

As a class of secondary metabolites of flavonoids, anthocyanins are the primary flower pigments in higher plants whose accumulations are tightly linked with flower development, making flowers show a series of colors, mainly ranging from red to purple and blue (Mol et al., 2008; Tanaka et al., 2008). Anthocyanin biosynthesis and accumulation are controlled by a complex environmental and developmental regulations (Weiss, 2000), such as UV light, nitrogen source, osmotic stress, and sugars. Among them, sugars are often used as the energy sources to improve the quality of cut flowers, such as lisianthus (*Eustoma grandiflorum*) (Jamal Uddin et al., 2001; Shimizu and Ichimura, 2005), oriental lily (*Lilium brownii*) (Han, 2003), and phlox (*Phlox paniculata*) (Sankhla et al., 2005), thereby indirectly improving the coloration of petals. However, the recent studies found that sugars not only serve as energy and structural materials in anthocyanin biosynthesis but also important signal molecules regulating its production (Rolland et al., 2002; Li et al., 2019). In *Arabidopsis*, higher anthocyanin accumulation was detected in the seedlings cultivated on sugar-containing plates, whereas the addition of sugar analogs had no such effect (Solfanelli et al., 2006). Similarly, exogenous sugars including glucose, fructose, or sucrose induced a large amount of anthocyanin production in detached hypocotyls of radish (*Raphanus sativus*) seedlings, but the osmotic control of 3OMG hardly caused anthocyanin accumulation (Hara et al., 2003). In addition, the cases that sugar enhanced anthocyanin accumulation through signal pathways were also found in radish hypocotyl (Hara et al., 2003), apple (*Malus domestica*) calli (Hu et al., 2016), petunia (*Petunia hybrida*) corolla (Moalem-Beno et al., 1997), and grape (*Vitis vinifera*) cells (Vitrac et al., 2000).

At present, the detailed information of sugars serving as signal molecules to regulate anthocyanin biosynthesis largely has not been revealed. A few studies suggested that this process was related to the regulation of gene expression levels involved in anthocyanin biosynthesis (Weiss, 2000; Rolland et al., 2002; Rook and Bevan, 2003; Mol et al., 2008; Naing et al., 2021). For example, in radish hypocotyl, the anthocyanin accumulation caused by sucrose treatment was related to higher transcripts of *CHS* and *ANS* (Hara et al., 2003). *CHS-A* from petunia was also a sugar-dependent expression gene in transgenic *Arabidopsis* (Tsukaya et al., 1991). In grape berry, research showed that sugar-induced anthocyanin accumulation was closely associated with incubation time and the RNA levels of F3H under different sugars (Zheng et al., 2009). Further transcriptome analysis suggested that sugar-induced anthocyanin production was not only related to the changed expression levels of structural genes but also the participation of regulatory genes, gathered with a massive modification in signaling pathways (Dai et al., 2013). Actually, acting upstream of structural genes involved in anthocyanin biosynthetic pathways (Dooner et al., 1991), transcription factors (TFs) are considered to be the important responders and performers in sugar-induced anthocyanin accumulation (Teng et al., 2005; Ai et al., 2016; Hu et al., 2016). In the recent years, more and more TFs that participate in sugar-induced anthocyanin production have been found and identified, including members of DELLA, WRKY, bHLH, MYB, and GARP families, and the MYB TFs are the most discovered and identified among them (Smeekens, 2000; Teng et al., 2005; Li et al., 2014; Hu et al., 2016; Chen et al., 2019; Zhao et al., 2021).

The R2R3-MYB transcription factor is the largest group of plant MYB factors that contain an R2R3 MYB domain at the N-terminal and an [DE]Lx_2_[RK]x_3_Lx_6_Lx_3_R motif in the R3 repeat for interacting with bHLH proteins, playing essential roles in the regulation of secondary metabolism including anthocyanin biosynthesis (Yan et al., 2021). To date, more and more R2R3-MYB TFs participating in the regulation of anthocyanin biosynthesis are discovered and identified in various fruit and flowers, such as cherry (*Prunus avium*) (Shen et al., 2014), apple (Jiang et al., 2014), lily (Yamagishi et al., 2010), and petunia (Quattrocchio et al., 1999). During flower color development, R2R3-MYBs are not only in charge of enzyme gene regulation but also responsible for the initiation of anthocyanin biosynthesis in response to a variety of developmental and environmental changes (Dooner et al., 1991). For instance, ABA-induced anthocyanin enhancement in sweet cherry was mediated by *PacMYBA* regulation (Shen et al., 2014). During the storage of kiwifruit (*Actinidia chinensis*), *AcMYBA1-1* and *AcMYB5-1* responded to low temperatures and induced the expression of several structural genes involved in anthocyanin biosynthesis (Li et al., 2017). More importantly, R2R3-MYB often appears in sugar response and plays a vital role in this process. For example, in *Arabidopsis*, *AtMYB75*, a key transcription factor for *DFR* activation, activated the anthocyanin biosynthesis when seedlings were exposed to high light or sucrose treatment (Teng et al., 2005; Li et al., 2016). *AtMyb56* regulated the anthocyanin accumulation by controlling the transcript level of *AtGPT2* in response to sucrose treatment (Jeong et al., 2018). Besides, *AtMYBL2* acted downstream of the transcription factor AtGLK1 and participated in *Arabidopsis* sucrose-induced anthocyanin biosynthesis (Zhao et al., 2021).

For the research on the anthocyanin biosynthesis in tree peony, many studies confirmed that it was mainly under the control of transcription factors including R2R3-MYB, bHLH, and WD40 repeats (Gu et al., 2019; Zhang et al., 2019; Qi et al., 2020). For example, PsMYB12 interacted with bHLH and WD40 protein to activate *PsCHS* expression, which was specific to the petal blotches (Gu et al., 2019). In flower color research of tree peony, people paid more attention to studies the such as double-color formation (Zhang et al., 2018), blotch formation (Zhang Y. et al., 2015; Gu et al., 2019), and petal pigmentation (Shi et al., 2015). However, the research on the effects of external factors such as light, temperature, and sugar on anthocyanin biosynthesis and their regulatory mechanism is insufficient. Previous study found that glucose treatment had a better color retention effect on *P. suffruticosa* ‘Luoyang Hong’ cut flower, and it promoted the anthocyanin accumulation of petals through signal pathways (Zhang C. et al., 2015). Research showed that the molecular mechanisms of petal coloration, as well as gene expression patterns involved in anthocyanin biosynthesis, varied among different anthocyanin compositions (Qian et al., 2021). As we know, *P. suffruticosa* ‘Luoyang Hong’ is a purple-red flower type cultivar with cyanidin (Cy)-dominated biosynthetic pathway in anthocyanin synthesis (Wang et al., 2001). Thus, whether glucose has the same pigmentation effect on pelargonidin (Pg)-based red pigment tree peony cut flowers remains to be determined.

In this study, *P. suffruticosa* ‘Tai Yang,’ belonging to pelargonidin (Pg)-based red pigment type, was selected as the material. The glucose and glucose analogs were applied to *P. suffruticosa* ‘Tai Yang’ cut flowers during vase time. The total anthocyanin (TA) content and individual anthocyanin were quantified through high-performance liquid chromatography (HPLC). The gene expression levels including nine structural genes and seven anthocyanin-related regulatory genes that maigt play positive role in glucose-induced anthocyanin production were detected to explore the possible regulation mechanism. Based on the comparison of gene expression between treatments, we found that the R2R3-MYB transcription factor *PsMYB2* showed a strong behavior to glucose treatment through the signal pathway in *P. suffruticosa* ‘Tai Yang’ cut flowers. The positive regulation effect of *PsMYB2* on anthocyanin biosynthesis was identified through transgenic tobacco. Additionally, the role of *PsMYB2* under glucose treatment was further studied in its transgenic *Arabidopsis* seedlings. These findings provided valuable data for elucidating the mechanism of glucose-induced anthocyanin biosynthesis and developing color retention technology for cut flowers.

## Materials and Methods

### Materials and Treatments

**FIGURE 1:**
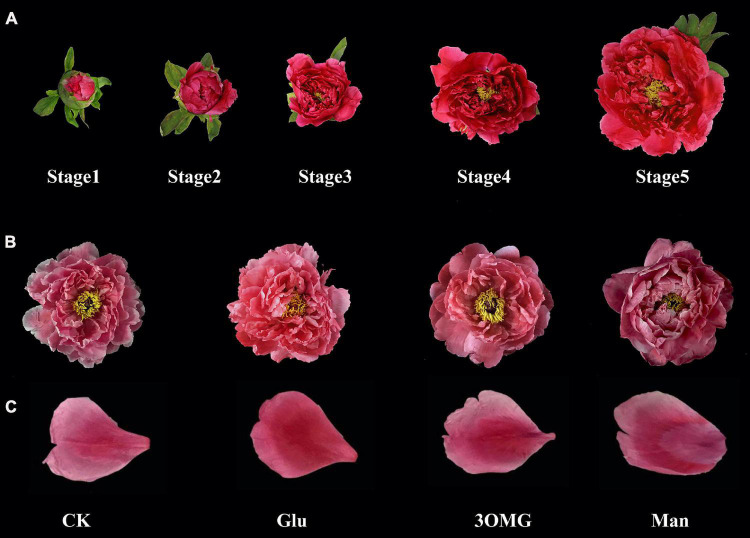
*P. suffruticosa* ‘Tai Yang’ **(A)** On-tree flowers of *P. suffruticosa* ‘Tai Yang’ at five developmental stages. Stage 1, soft bud stage; stage 2, pre-opening stage; stage 3, initial opening stage; stage 4, half opening stage; stage 5, full opening stage. **(B)** Flowers at stage 5 under four treatments. **(C)** Petals of flowers under four treatments. There were four treatments including CK, Glu, 3OMG, and Man, respectively. CK was the control group that cut flowers treated with distilled water. Glu, 3OMG, and Man were the cut flowers treated with glucose, 3-O-methylglucose, and mannose at 333 mM, respectively.

Tree peony (*P. suffruticosa* ‘Tai Yang’) used in this study was cultivated in peony planting base in Heze, Shandong Province, China. Referring to our previous study (Guo et al., 2004), flowers that developed to stage 1 (S1, soft bud stage) were harvested, with flower branches about 30 cm in length and upper compound leaves retained. After harvesting, flowers were transported to the laboratory within 12 h. Before further treatment, all flower branches were retrimmed to 25 cm length with leaves removed, and then, the flower’s stems were placed into distilled water for 1 h for water recovery. After that, the flowers at S1 were randomly divided into four groups and inserted into glass bottles added with 100 ml of different holding solutions. The holding solution with distilled water only was the control (CK, in vase flowers), and the distilled water containing glucose (Glu), 3-O-methylglucose (3OMG, as osmotic control), and mannose (Man, for HXK-depend signaling pathway detecting), respectively, were the other three treatments. According to previous research that 333 mM glucose had a good color retention effect on tree peony cut flowers (Zhang C. et al., 2015), the concentration of glucose, 3OMG, and mannose in this study was set to 333 mM. Besides, all solutions contained 0.05% (v/v) NaClO to prevent the growth of bacteria. There were a total of thirty cut flowers per treatment. The vase condition was maintained at room temperature at 20–23°C and relative humidity of 50–60%, under 40 μmol m^–2^s^–1^ light intensity with 12-h photoperiod. The solutions or distilled water were replaced every day at fixed times. Referring to the opening stages given in Figure 1A (stage 2, S2, pre-opening stage; stage 3, S3, initial opening stage; stage 4, S4, half opening stage; and stage 5, S5, full opening stage), the middle petals of flowers at four developmental stages under different treatments were sampled and quickly frozen in liquid nitrogen. All samples were stored at –80°C for anthocyanin determination and gene expression analysis. In addition, the flowers that grew naturally in the field condition and opened to S5 were sampled for anthocyanin compositions analysis of *P. suffruticosa* ‘Tai Yang’ flowers.

Plants of *Nicotiana benthamiana* and *Nicotiana tabacum* used in the experiment were cultivated for about 4–8 weeks at a 22°C chamber with 14-h light/10-h dark condition and 60–70% relative humidity. Then, they were selected for the transient expression analysis and stable transformation materials by *Agrobacterium tumefaciens*, respectively.

*Arabidopsis* for genetic transformation were the Columbia ecotype (Col-0). The seedlings were grown under a 16-h/8-h (light/dark) cycle at 23/21°C until required.

### Petal Color Measurement

Flower surface color was assessed using the Spectrophotometer NF333 (Nippon Denshoku Industries Co., Ltd., Japan). The color reading was taken five times from the upper epidermis region of middle petals (the 4th–6th layer petals) and then averaged as one replicate. A total of three replicates were required for each flower color evaluation. The color measurements at every developmental stage (S2–S5) for each treatment were generated from five single cut flowers. The flower color values of L* (lightness), a* (from green to red), and b* (from blue to yellow) were generated under daylight conditions with the instrument. In addition, the C* (chroma) value was calculated with the formula of C* = (a^*2^ + b^*2^)^0.5^ (Voss, 1992).

### Anthocyanin Content Measurement

For anthocyanin composition analysis of *P. suffruticosa* flowers, the petals of each sample with about 0.3 g fresh weight were extracted with 5 ml extraction solvent (CH_3_OH: HCl: H_2_O = 70: 0.1: 29.9; v/v/v) at 4°C in the dark for 24 h, shaking the mixture with a vortex per 8 h during this period. The contents and changes of anthocyanin composition in the tree peony petals under four treatments were qualified with the method of HPLC according to a protocol reported by Zhang et al. (2018) with a few modifications. First, the supernatants were filtered with a 0.22-μm syringe filter and analyzed on an Agilent 1100 High Performance Liquid Chromatograph (Agilent, United States). The mobile phase (A) was 1% formic acid, and the mobile phase (B) was 15% methanol acetonitrile. The program parameter was set as follows: 0 min, 5% B; 42 min, 8% B; and 45 min, 8% B. The injection volume was 10 μl, and the flow rate was 1.0 ml min^–1^. The C18 column of ODS-80Ts QA (4.6 and 150 mm) (Tosoh, Japan) was used for anthocyanin separating with a column temperature of 35°C at 515 nm.

The TA content analysis of tobacco flowers and *Arabidopsis* seedlings were measured with the spectrophotometric method. Samples of tobacco flowers and *Arabidopsis* seedlings with approximately 100 and 20 mg, respectively, were incubated in 1% HCl/methanol solution in the darkness at 4°C for 24 h. The absorbance of the supernatant was monitored at 530 and 657 nm, and the TA contents were calculated with the formula of (A530−0.25 × A657) × M^–1^. M was the fresh weight (g) of the samples. There were three biological replicates for each sample.

### Soluble Sugar Content Measurement

The frozen petals of *P. suffruticosa* flower under four treatments with approximately 0.2 g for each sample were ground in liquid nitrogen and extracted with 80% ethanol (v/v) in 80°C water bath for 30 min. The mixture was centrifuged at 10,000 × *g* for 5 min at room temperature, and the supernatant was collected into the volumetric flask. This extraction step was repeated two times. The soluble sugar content was measured with anthrone-sulfuric acid at a wavelength of 620 nm (Hedge et al., 1962).

### Total RNA Extraction, cDNA Synthesis, and Gene Expression Analysis

Total RNAs were respectively extracted from tissues of *P. suffruticosa*, tobacco, and *Arabidopsis* using the EASY Spin Plant RNA Rapid Extraction Kit (Aidlab, China) according to the manufacturer’s instructions. The concentration and quality were evaluated with a NanoDrop 2000C spectrophotometer (NanoDrop, United States). Subsequently, RNase-free DNase I (Takara, Japan) was used to remove the potential DNA contamination in RNA. About 1 μg of total RNA was used to synthesize the cDNA using M-MLV (Takara, Japan) according to the manufacturer’s protocol.

Real-time PCRs for *P. suffruticosa*, tobacco, and *Arabidopsis* were carried out on a CFX96 real-time PCR machine (Bio-Rad, United States) in a total reaction mixture of 20 μl in each containing 10 μl of SYBR Green Premix Ex Taq TM (TaKaRa, Japan), 1 μl of each primer (10 mM), 2 μl of diluted cDNA, and 6 μl of ddH_2_O. The qPCR amplification protocol parameter was as follows: 30 s at 95°C, followed by 40 cycles of 5 s at 95°C, 30 s at 62°C, and 30 s at 72°C, followed by 65 to 95°C melting curve detection. Primers for the individual genes are listed in Supplementary Table 1. The internal control gene of qPCR assay in *P. suffruticosa*, tobacco, and *Arabidopsis* were *Psubiquitin*, *NtTub1*, and *AtActin2*, respectively. Relative gene expression levels were obtained *via* the 2^–ΔΔ*Ct*^ method. Each measurement was taken with three biological replicates.

### Cloning, Sequence, and Subcellular Localization Analysis of *PsMYB2*

In our previous study, an Illumina/Solexa library of *P. suffruticosa* ‘Luoyang Hong’ flower petal was constructed and sequenced. A gene (GenBank accession number: KJ466975) encoding R2R3-MYB transcription factor PsMYB2 was obtained with reverse transcriptase-polymerase chain reaction (RT-PCR), and the open reading frame (ORF) was identified in our previous study (Zhang et al., 2014). The complete coding region of *PsMYB2* was amplified from the cDNA templates of *P. suffruticosa* ‘Tai Yang’ using PrimeSTAR HS DNA Polymerase (Takara, Japan). Primer sequences used for *PsMYB2* cloning are shown in Supplementary Table 1. Multiple sequence alignment was generated by the DNAMAN software version 6. The phylogenetic tree was constructed with MYB TFs from other species using the neighbor-joining method of MEGA11 software, and the tree nodes were evaluated with 1,000 bootstrap replicates.

For subcellular localization analysis, the coding region of *PsMYB2* was cloned to a pBI121-GFP vector, forming a pBI121-GFP-*PsMYB2* construct. Primer sequences used for vector construction are listed in Supplementary Table 1. The plasmids of pBI121-GFP-*PsMYB2* and empty pBI121-GFP were transformed into the GV3101 strain and then were transiently expressed in leaves of 4 to 6-week-old tobacco plants *via* agrobacterium injection. The green fluorescent protein (GFP) signals were detected with a fluorescence microscope (TCS SP8, Leica, Germany) after 48–72 h of infiltration.

### Tobacco and *Arabidopsis* Stable Transformation

To detect the function of *PsMYB2*, the coding region of *PsMYB2* was cloned to the pBI121 vector driving by the CaMV35s promoter, constructing a pBI121-*PsMYB2* overexpression vector. Primer sequences used for vector construction are shown in Supplementary Table 1. Then, the pBI121-*PsMYB2* plasmid was stably transferred into tobacco and *Arabidopsis* by leaf disk method (Horsch et al., 1985) and feather dip method (Clough and Bent, 1998), respectively. Transformed tobacco plants and *Arabidopsis* seedlings were selected using kanamycin (100 mg ml^–1^) as a selective marker, and the transgenic lines hosting *PsMYB2* were further identified by PCR amplification assay (Supplementary Figures 1A,B). A total of three independent T2 progeny *PsMYB2*-overexpressing tobaccos and two independent homozygous T3 *Arabidopsis* seeds with higher *PsMYB2* gene expression levels were selected for further analysis.

### *Arabidopsis* Treatment With Glucose

Homozygous T3 seeds of transgenic *PsMYB2 Arabidopsis* were plated on the one-half-strength MS medium with 0.7% plant agar, pH 5.8, including vitamins. The culture medium added with 100 mM glucose was the treatment group, and no sugar added was the control group (CK). In addition, the culture medium added with 100 mM 3OMG was used as the osmotic control group (3OMG). About 100 seeds were sowed for each treatment. The seeds were cultured on plates at 4°C in the dark for 4 days. Then, the seeds were cultured at 22°C under continuous fluorescent light. After 5 days of growth, seedlings were harvested and soon frozen in the liquid nitrogen. Then, all samples were stored at –80°C for TA content measurement and gene expression analysis.

### Statistical Analysis

Statistical data were calculated with software SPSS version 20.0 (IBM, NY, United States). All values were shown as the mean ± standard errors at least three replicates. Data differences in flower color, soluble sugar content, anthocyanin content, and gene expression levels of *P. suffruticosa* ‘Tai Yang’ cut flowers were calculated through Duncan’s multiple range test at 0.05 probability.

## Results

### The Flower Color Changes of Four Treatments at Different Developmental Stages in *P. suffruticosa* ‘Tai Yang’

**FIGURE 2:**
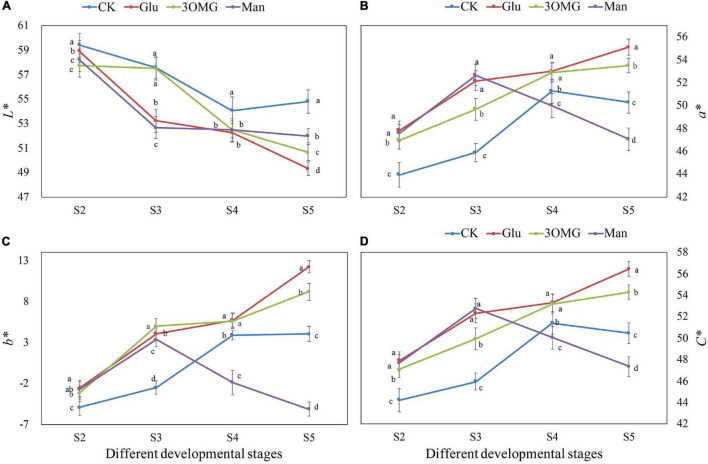
The flower color values of *P. suffruticosa* ‘Tai Yang’ cut flowers under four treatments. **(A)** Lightness (L*). **(B)** Redness (a*). **(C)** Yellowness (b*). **(D)** Chroma (C*). There were four treatments including CK, Glu, 3OMG, and Man, respectively. CK was the control group that cut flowers treated with distilled water. Glu, 3OMG, and Man were the cut flowers treated with glucose, 3-O-methylglucose, and mannose at 333 mM, respectively. S2, pre-opening stage; S3, initial opening stage; S4, half opening stage; S5, full opening stage. Each value was expressed as the mean ± standard errors (SE) of triplicate replications. Different small letters indicated significant differences among treatments at the same developmental stage according to Duncan’s multiple range test at *p* < 0.05.

As shown in Figures 1B,C, the flower color under the glucose treatment was more flamboyant with the naked eye, and the coloration of the middle petal under glucose treatment was much uniform than other treatments. The petal color changes at four developmental stages under different treatments are shown in Figure 2. The petal color of cut flowers was presented as lightness (L*), redness (a*), yellowness (b*), and chroma (C*). Compared with the CK, the L* value of the cut flowers was decreased by the glucose, 3OMG, and mannose treatments during the whole flower opening process, except for no difference of 3OMG treatment at S3. At the full opening stage (S5), L* values were reduced by 10, 8, and 5% under glucose, 3OMG, and mannose treatments, respectively, compared with the CK treatment (Figure 2A).

The a* value changing trend of cut flowers under four treatments varied, a* value under CK and mannose treatments increased first, reaching the highest level at S4 and S3, respectively, and then declined (Figure 2B). In contrast, it remained to increase from S2 to S5 under glucose and 3OMG treatments. During the entire opening stages, the a* value of glucose and 3OMG treatment continued to be higher than CK. The a* value of mannose treatment was higher than that of the CK at the early stages (S2–S3) and then became lower than CK due to the continuous decline in the later stages (S4-S5). At the full opening stage (S5), the a* value of glucose treatment was 55.12, which was the highest among the four treatments, followed by 3OMG, CK, and mannose treatments with the values of 53.50, 50.28, and 47.07, respectively (Figure 2B). Besides, the b* and C* value changing trends were the same as a* values in four treatments (Figures 2C,D). At the full opening stage, the glucose treatment owned the highest value of b* among four treatments, followed by 3OMG, CK, and mannose (Figure 2C). Similarly, C* value of glucose treatment at S5 was the highest with the value of 56.47, which was 4, 12, and 19% higher than 3OMG, CK, and mannose, respectively (Figure 2D).

### Total Soluble Sugar and Anthocyanin Contents of Flowers at Different Developmental Stages

**FIGURE 3:**
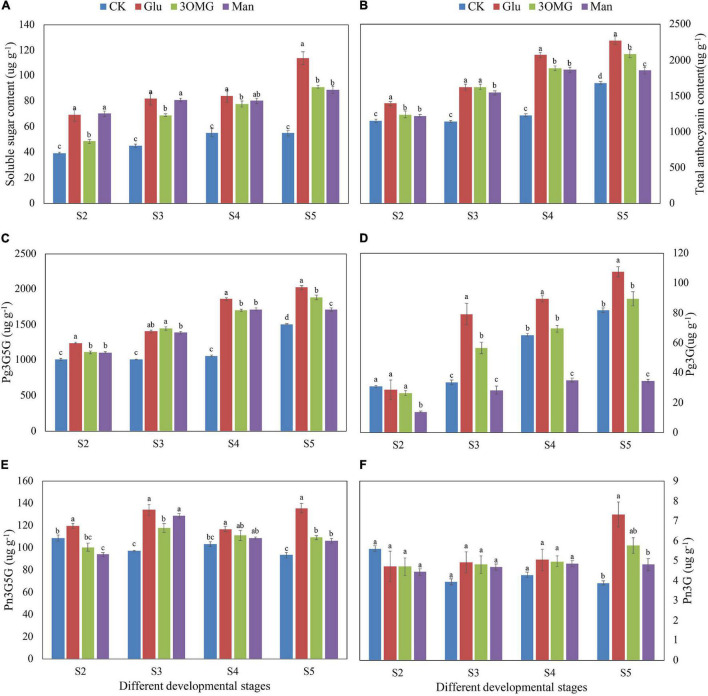
The soluble sugar contents in petals of *P. suffruticosa* ‘Tai Yang’ cut flowers under four treatments **(A)**. The total anthocyanin contents in petals of *P. suffruticosa* ‘Tai Yang’ cut flowers under four treatments **(B)**. The contents of four anthocyanin compositions in the petals of *P. suffruticosa* ‘Tai Yang’ cut flowers under four treatments **(C–F)**. **(C)** Pelargonidin-3,5-di-O-glucoside (Pg3G5G); **(D)** pelargonidin 3-O-glucoside (PG3G); **(E)** peonidin-3,5-di-O-glucoside (Pn3G5G); **(F)** peonidin-3-O-glucoside (Pn3G). There were four treatments including CK, Glu, 3OMG, and Man, respectively. CK was the control group that cut flowers treated with distilled water. Glu, 3OMG and Man were the cut flowers treated with glucose, 3-O-methylglucose and mannose at 333 mM, respectively. S2, pre-opening stage; S3, initial opening stage; S4, half opening stage; S5, full opening stage. Each value was expressed as the mean ± standard errors (SE) of triplicate replications. Different small letters indicated significant differences among treatments at the same developmental stage according to Duncan’s multiple range test at *p* < 0.05.

The soluble sugar content in four treatments showed an upward trend with the opening of cut flowers (Figure 3A). In the CK flowers, soluble sugar content increased with the flowers opening, reaching a maximum value of 55.16 ug g^–1^ at S4, and then no longer increased. However, compared with the CK, the soluble sugar content of cut flowers under the glucose, 3OMG, and mannose treatments continued to increase from S2 to S5, showing greater increases than that of the CK at all corresponding stages (Figure 3A). At the early opening stages (S2–S3), the soluble sugar contents under the glucose and mannose treatments were about 1.80 times that of the CK treatment, which was higher than that of the 3OMG treatment. During the late opening stages (S4–S5), mannose’s enhancement on sugar content became weaker with no difference with 3OMG treatment. In contrast, sugar content under glucose treatment was still remained at the highest level among four treatments. At the full opening stage (S5), the soluble sugar contents under glucose, 3OMG, mannose, and CK were 113.46 > 90.21 > 88.99 > 54.84 ug g^–1^, respectively.

The changes in TA content in petals under four treatments were similar to that of soluble sugar content, with a gradually increasing trend overall during the flowers opening (Figure 3B). Compared with the CK treatment, the TA content in petals showed greater increases under glucose, 3OMG, and mannose treatments at corresponding stages. Among four treatments, glucose treatment made the highest TA content, higher than 3OMG, mannose, and CK, except for S3 with no significance with 3OMG. In addition, TA content under 3OMG treatment was higher than that of mannose, but it did not reach significance in S2 and S4 stages. When the cut flowers opened to the best viewing period (S5), the TA content order of petals was glucose > 3OMG > Man > CK, with the content of 2,276.47, 2,087.29, 1,855.62, and 1,684.42 ug g^–1^, respectively.

### Qualitative and Quantitative Analysis of Anthocyanin Changes at Different Developmental Stages in *P. suffruticosa* ‘Tai Yang’ Under Four Treatments

First, the anthocyanin compositions of *P. suffruticosa* ‘Tai Yang’ flowers were detected. According to HPLC data analysis, there were four anthocyanin compositions in the petals of the flowers at S5 that grew naturally in the field condition, including pelargonidin-3-O glucoside (Pg3G), pelargonidin-3,5-di-O-glucoside (Pg3G5G), peonidin-3-O-glucoside (Pn3G), and peonidin-3,5-di-O-glucoside (Pn3G5G). Among them, Pg3G5G had the highest proportion of 69%, followed by Pg3G, Pn3G5G, and Pn3G with the ratios of 25, 5, and 1%. The results are shown in Supplementary Table 2. During postharvest development, four anthocyanin compositions’ changes in cut flower petals under different treatments were as follows:

As the main anthocyanin composition of *P. suffruticosa* ‘Tai Yang’ flowers, in the CK flowers, with the opening of cut flowers, Pg3G5G content increased and reached the highest level at the full opening stage (S5) (Figure 3C). Compared with the CK treatment, glucose, 3OMG, and mannose treatments showed greater increases. But Pg3G5G content under the glucose treatment was much greater than other treatments at the corresponding stages, except for S3. At the full opening stage, Pg3G5G content was 2,025.72 ug g^–1^ in glucose, which was 1.35, 1.08, and 1.18 times that of the CK, 3OMG, and mannose treatments, respectively (Figure 3C).

In the CK flowers, Pg3G content showed a linear upward trend from S2 to S5, reaching the maximum at S5 with the value of 81.66 ug g^–1^, which was 50.97 ug g^–1^greater than that of the pre-opening stage (S2) (Figure 3D). Compared with the CK flowers, Pg3G content in glucose was greatly enhanced during the later developmental stages (S3–S5) (Figure 3D). When flowers fully opened (S5), Pg3G content reached the highest level with the value of 107.58 ug g^–1^, which was 1.32 times that of the control at S5 (Figure 3D). In the 3OMG treatment, Pg3G content from S3 to S5 was also higher than CK, but the difference was only significant at S3 (Figure 3D). Unlike glucose and 3OMG treatments, mannose inhibited the increase in Pg3G content, and Pg3G content was much lower than that of CK from S2 to S5, except for no difference at S3 (Figure 3D).

With the opening of cut flowers, Pn3G5G content was unstable in CK flowers, showing a descending trend overall from S2 to S5. At full opening stage (S5), Pn3G5G content of CK was 93.46 ug g^–1^, which was 15.45 ug g^–1^ less than that of the pre-opening stage (S2) (Figure 3E). Compared with the CK flowers, Pn3G5G content was increased by the glucose treatment, which was higher than CK from S2 to S5. Specifically, at S5, Pn3G5G content (135.85 ug g^–1^) under glucose treatment was 1.45 times that of the CK flowers (Figure 3E). In addition, 3OMG and mannose treatments also enhanced the accumulation of Pn3G5G but were both lower than that of the glucose treatment (Figure 3E).

The content of Pn3G was the least in *P. suffruticosa* ‘Tai Yang’ flowers. With the opening of flowers, Pn3G content in the CK decreased from S2 to S3 and then remained stable from S3 to S5 (Figure 3F). As Figure 3F shows, from S3 to S4, although the Pn3G contents of glucose, 3OMG, and mannose treatments were higher than that of the CK, they did not reach the statistical significance. However, at the full opening stage (S5), Pn3G content under the glucose treatment was the highest with the value of 7.32 ug g^–1^, which was higher than that of the mannose and CK treatments (Figure 3F).

### Gene Expression Changes of Nine Structural Genes at Different Developmental Stages in *P. suffruticosa* ‘Tai Yang’ Under Four Treatments

**FIGURE 4:**
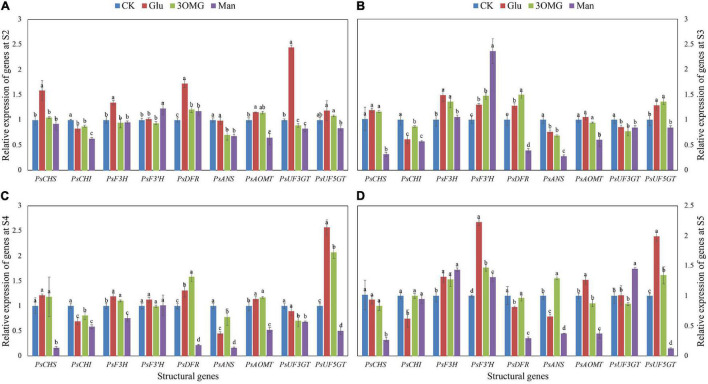
Relative expression levels of nine anthocyanin biosynthetic structural genes in petals of *P. suffruticosa* ‘Tai Yang’ cut flowers under four treatments. **(A)** S2, pre-opening stage; **(B)** S3, initial opening stage; **(C)** S4, half opening stage; **(D)** S5, full opening stage. There were four treatments including CK, Glu, 3OMG, and Man, respectively. CK was the control group that cut flowers treated with distilled water. Glu, 3OMG, and Man were the cut flowers treated with glucose, 3-O-methylglucose, and mannose at 333 mM, respectively. Each value was expressed as the mean ± standard errors (SE) of triplicate replications. Different small letters indicated significant differences among treatments at the same developmental stage according to Duncan’s multiple range test at *p* < 0.05.

The abundance of structural gene expression will directly affect the amount of anthocyanin biosynthesis and composition. Gene expression levels of nine structural genes at different developmental stages under four treatments are shown in Figure 4. Compared with the CK treatment, at S2 (Figure 4A), more than half of the genes, including *PsCHS*, *PsF3H*, *PsDFR*, *PsAOMT*, and *PsUF3GT*, were upregulated by glucose. However, 3OMG only showed an upregulation effect on *PsDFR* and *PsAOMT*. For mannose treatment, it showed a promotion effect on *PsF3′H* and *PsDFR.* Moreover, the *PsF3′H* expression level of mannose was higher than that of other treatments. With the development of flowers, at the initial opening stage (S3) (Figure 4B), glucose continued to upregulate the expression of *PsF3H* and *PsDFR* genes, and the expression of *PsF3′H* and *PsUF5GT* also began to be promoted by glucose. At this stage, the genes upregulated by 3OMG treatment were the same as glucose treatment, and the *PsDFR* expression level under 3OMG treatment was higher than glucose. Compared with the CK, although the mannose treatment showed inhibition on most genes at S3, it continued to favor the induction on the *PsF3′H* gene expression, which was higher than glucose and 3OMG, with about 0.82 and 0.60 times higher than theirs, respectively. The genes upregulated by glucose and 3OMG treatment were still the same at S4 (Figure 4C), including *PsF3H*, *PsDFR*, *PsAOMT*, and *PsUF5GT*. The *PsUF5GT* expression level under glucose treatment was higher than that of 3OMG, whereas *PsDFR* was the opposite. At the same stage, except for *PsF3′H*, the expression levels of the other eight structural genes were all inhibited by mannose treatment (Figure 4C). The full opening stage (S5) was the best viewing period for tree peony cut flowers, where the cut flowers were kept for the longest time. During this period, genes including *PsF3H*, *PsF3′H*, *PsAOMT*, and *PsUF5GT* showed high expression levels under glucose treatment (Figure 4D). Although several genes were promoted by 3OMG treatment at this stage, its regulation effect was not as good as glucose treatment overall. For example, the expression of *PsF3′H* and *PsUF5GT* under glucose treatment was about 1.52 and 1.48 times of 3OMG treatment, respectively (Figure 4D). For mannose treatment, the genes inhibited by mannose treatment reduced at S5. Furthermore, the expression levels of *PsF3H*, *PsF3′H*, and *PsUF3GT* were promoted by mannose (Figure 4D).

### Gene Expression Changes of Seven Regulatory Genes at Different Developmental Stages in *P. suffruticosa* ‘Tai Yang’ Under Four Treatments

**FIGURE 5:**
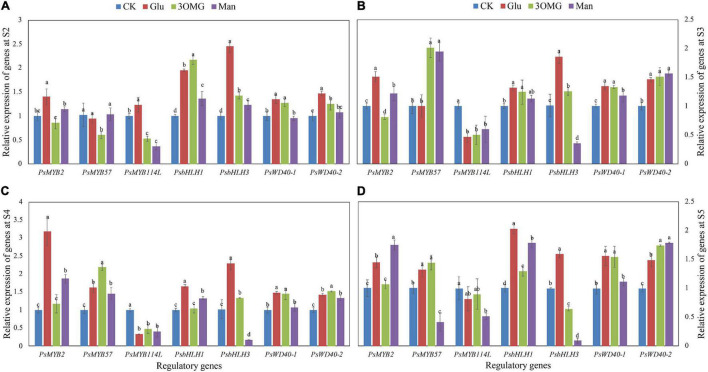
Relative expression levels of seven anthocyanin biosynthetic regulatory genes in petals of *P. suffruticosa* ‘Tai Yang’ cut flowers under four treatments. **(A)** S2, pre-opening stage; **(B)** S3, initial opening stage; **(C)** S4, half opening stage; **(D)** S5, full opening stage. There were four treatments including CK, Glu, 3OMG, and Man, respectively. CK was the control group that cut flowers treated with distilled water. Glu, 3OMG, and Man were the cut flowers treated with glucose, 3-O-methylglucose, and mannose at 333 mM, respectively. Each value was expressed as the mean ± standard errors (SE) of triplicate replications. Different small letters indicated significant differences among treatments at the same developmental stage according to Duncan’s multiple range test at *p* < 0.05.

The expression of structural genes during anthocyanin biosynthesis is directly controlled by regulatory genes. In this study, seven regulatory genes, including three MYB TFs, two bHLH TFs, and two WD40 TFs, were monitored during postharvest development of flowers. Compared with CK treatment, except for *PsMYB57* and *PsMYB114L*, all genes, including *PsMYB2*, *PsbHLH1*, *PsbHLH3*, *PsWD40-1*, and *PsWD40-2*, continuously showed high expression levels under glucose treatment from S2 to S5 (Figure 5). Specifically, in *PsMYB2*, *PsbHLH1*, and *PsbHLH3*, their expression levels at the corresponding stages were 1.30–2.40 times that of the CK. Among these five genes continuously induced by glucose, *PsWD40-1* and *PsWD40-2* showed a sustained high expression state under 3OMG treatment (Figure 5). Additionally, the *PsWD40-2* expression level under 3OMG was greater than glucose at late opening stages (S4–S5) (Figures 5C,D). Although the gene expression levels of *PsbHLH1* at S3 and *PsbHLH3* at S5 under 3OMG treatment had no difference with CK treatment, they were higher than that of CK treatment at the other three stages (Figure 5). The expression level of *PsbHLH1* at S2 was especial, which was 2.18 times that of the CK treatment and higher than that of glucose treatment (Figure 5A). Besides, the expression level of *PsbHLH3* gene was lower than that of glucose in all stages (Figure 5). The expression levels of these five regulatory genes under mannose were lower than those of glucose and 3OMG treatments overall. Still, the genes including *PsMYB2*, *PsbHLH1*, and *PsWD40-2* were upregulated by mannose treatment in the most stages (at least 3 stages), compared to CK treatment (Figure 5). For example, except for no difference with the CK treatment at the pre-opening stage (S2), *PsMYB2* continued to be highly expressed from S3 to S5 under mannose treatment (Figures 5B–D), and especially at the S5, its expression level was higher than that of glucose and 3OMG treatments with 0.75 times higher than that of the CK (Figure 5D). It seemed that glucose treatment had a weaker promotion effect on *PsMYB57* and *PsMYB114L*. Compared with the CK, *PsMYB57* was only highly expressed at the S4 to S5 stages under glucose treatment, and the expression level of *PsMYB114L* was inhibited by glucose treatment at S3 to S4 stages (Figures 5B,C). Although 3OMG treatment also inhibited *PsMYB114L* expression level at late opening stages (S3–S5), *PsMYB57* expression was enhanced by 3OMG treatment, which was 2.01, 2.19, and 1.45 times that of the CK at S3, S4, and S5, respectively. In addition, although the expression level of *PsMYB114L* was continuously downregulated by mannose treatment throughout four opening stages (S2–S5) (Figure 5), *PsMYB57* was highly expressed under mannose treatment during the middle opening stages (S3–S4) (Figures 5B,C).

Through the expression analysis of regulatory genes under four treatments, both mannose and glucose showed the promotion effects on *PsMYB2*, *PsbHLH1*, and *PsWD40-2*. Among these three genes, *PsbHLH1* and *PsWD40-2* were also upregulated by 3OMG treatment, whereas only *PsMYB2* was hardly induced by 3OMG treatment (Figure 5). Thus, we speculated that *PsMYB2* might be an essential transcription factor in glucose inducing anthocyanin biosynthesis of *P. suffruticosa* ‘Tai Yang’ cut flowers through the HXK-dependent signaling pathway. Therefore, we conducted further research on *PsMYB2*.

### Sequence and Subcellular Localization Analysis of *PsMYB2*

**FIGURE 6:**
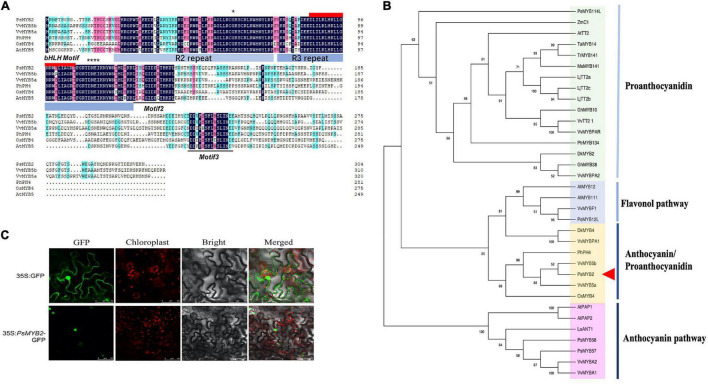
Sequence analyses of *PsMYB2*. **(A)** Protein sequence alignment analysis of the PsMYB2. Sequence alignment was performed using the DNAMAN software of version 6. GenBank accession numbers of these MYB proteins were as follows: VvMYB5b, *Vitis vinifera*, AAX51291.1; VvMYB5a, *Vitis vinifera*, AAS68190.1; PhPH4, *Petunia hybrida* AAY51377.1; OsMYB4, *Oryza sativa*, BAA23340.1; AtMYB5, *Arabidopsis thaliana*, NP_187963.1. **(B)** Phylogenetic analysis of PsMYB2 with MYB transcription factors from other species. The phylogenetic tree was constructed with MEGA11 software through neighbor-joining method and the tree nodes were evaluated with 1000 bootstrap replicates. GenBank accession numbers of the MYB proteins were as follows: ZmC1, *Zea mays*, AAA33482.1; AtTT2, *Arabidopsis thaliana*, Q9FJA2.1; LjTT2c, *Lotus japonicus*, BAG12895.1; LjTT2b, *Lotus japonicus*, BAG12894.2; LjTT2a, *Lotus japonicus*, BAG12893.1; TaMYB14, *Trifolium arvense*, AFJ53053.1; TrMYB141, *Trifolium repens*, AFJ53048.1; MsMYB141, *Medicago sativa*, AFJ53055.1; GhMYB10, *Gossypium hirsutum*, NP_001314513.1; PtrMYB134, *Populus trichocarpa*, XP_002308528.1; VvTT2_1, *Vitis vinifera*, RVW44110.1; VvMYBPAR, *Vitis vinifera*, BAP39802.1; DkMYB2, *Diospyros kaki*, BAI49719.1; GhMYB38, *Gossypium hirsutum*, AAK19618.1; VvMYBPA2, *Vitis vinifera*, ACK56131.1; AtMYB12, *Arabidopsis thaliana*, NP_182268.1; AtMYB111, *Arabidopsis thaliana*, NP_199744.1; VvMYBF1, *Vitis vinifera*, ACT88298.1; PsMYB12L, *Paeonia suffruticosa*, QBK15080.1; DkMYB4, *Diospyros kaki*, BAI49721.1; VvMYBPA1, *Vitis vinifera*, CAJ90831.1; PhPH4, *Petunia hybrida*, AAY51377.1; VvMYB5b, *Vitis vinifera*, AAX51291.1; VvMYB5a, *Vitis vinifera*, AAS68190.1; OsMYB4, *Oryza sativa*, BAA23340.1; PsMYB58, *Paeonia suffruticosa*, QZJ84669.1; AtPAP1, *Arabidopsis thaliana*, AAG42001.1; AtPAP2, *Arabidopsis thaliana*, NP_176813.1; SlANT1, *Solanum lycopersicum*, AAQ55181.1; PsMYB57, *Paeonia suffruticosa*, QIG55740.1; VvMYBA2, *Vitis vinifera*, BAD18978.1; VvMYBA1, *Vitis vinifera*, BAD18977.1. **(C)** The subcellular localization of *PsMYB2*. 35S: GFP, control group; 35S: *PsMYB2*-GFP, PBI121-GFP-*PsMYB2*.

First, the full-length cDNA sequence of *PsMYB2*, encoding an R2R3-MYB protein, was obtained by PCR from a tree peony cDNA library of *P. suffruticosa* ‘Tai Yang.’ The sequencing result indicated that it had a 918 bp ORF encoding a protein of 305 amino acids. Multiple sequence alignment of amino acids showed that it contained a conserved R2R3 DNA-binding domain at the N-terminus and a bHLH interaction motif ([D/E]Lx_2_[R/K]x_3_Lx_6_Lx_3_R) in the R3 domain (Figure 6A). In addition, two specific motifs were found in the C-terminal variable region of *PsMYB2*: Motif 2 and Motif 3 (Figure 6A). Motif 2 was called C1 (Lx_3_GIDPxTHKPL), found in the proteins belonging to the MYB subfamily 4, and initially characterized by Kranz et al. (1998). Another one was Motif 3 and temporarily named C3 (DDxF[S/P] SFL[N/D]SLIN[E/D]). This conserved motif was only found in a few MYB protein sequences gathered to the *VvMYB5a/b* cluster. Besides, Gly and DNEI (Asp–Asn–Glu1–Ile), in the R2 and R3, respectively, were found in PsMYB2 protein, which was related to the biosynthesis of anthocyanin and proanthocyanidin (Heppel et al., 2013). Finally, no C2 motif was found in the PsMYB2 protein (Jin et al., 2000).

To better understand the feature of *PsMYB2*, R2R3-MYBs associated with different functions were selected for phylogenetic analysis with the neighbor-joining method. The result showed that the R2R3-MYBs were robustly separated into 4 sub-branches with more than 60% bootstrap percentages (Figure 6B). Among 34 R2R3-MYBs, PsMYB2 was gathered into VvMYB5a/b cluster with 98% bootstrap percentages of this cluster, indicating that the VvMYB5a/b sub-branch was very stable. Based on the different roles in the flavonoid biosynthetic pathway, they were named as flavonol, anthocyanin, anthocyanin/proanthocyanidin, and proanthocyanidin, respectively (Figure 6B). PsMYB2 was grouped into the clade of anthocyanin/proanthocyanidin and closed to the VvMYB5a and VvMYB5b, which were positively associated with anthocyanin and proanthocyanidin production (Deluc et al., 2006, 2008). Sequence similarity showed that the PsMYB2 protein sequence displayed 55 and 50% identical to VvMYB5b and VvMYB5a, respectively.

Furthermore, the subcellular localization of *PsMYB2* was also detected. Both recombinant vector PBI121-GFP-*PsMYB2* and the control vector (PBI121-GFP) were transiently expressed into the *Nicotiana benthamiana* leaves. The results showed that GFP fluorescence was observed in the nucleus and cytomembrane in the control group, whereas the group with the PBI121-GFP-*PsMYB2* vector only showed fluorescence signals in the nucleus (Figure 6C). Thus, we speculated that the *PsMYB2* transcription factor was localized and functioned in the nucleus.

### Overexpression of *PsMYB2* in Tobacco Promotes the Anthocyanin Accumulation of Tobacco Flowers

**FIGURE 7:**
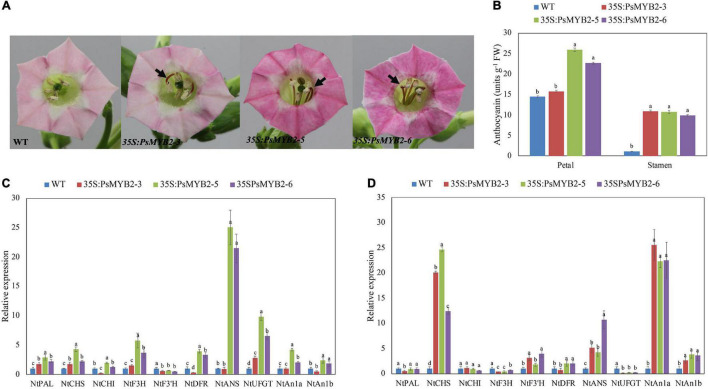
Overexpression of *PsMYB2* in tobacco. **(A)** Overexpression of *PsMYB2* increased the anthocyanin accumulation in tobacco flowers. Flowers of transgenic tobacco showed increased pigmentation in petal and stamen, compared to wild-type flowers. **(B)** The TA content in the petal and stamen of the transgenic flowers was higher than that of the wild type. **(C,D)** Were the gene expression levels related to anthocyanin biosynthesis in the petal and stamen of tobacco flowers, respectively. Each value was expressed as the mean ± standard errors (SE) of triplicate replications. Different small letters indicated significant differences among *PsMYB2* transgenic lines and wild-type plants according to Duncan’s multiple range test at *p* < 0.05. WT, wild-type plant; *35S:PsMYB2* (3, 5, and 6), plant overexpressing *PsMYB2.*

To verify its function on anthocyanin biosynthesis, we constructed a *PsMYB2* overexpression vector and stably transformed it into tobacco. As shown in Figure 7A, the darker flower petals were visually observed in tobacco line 5 and 6 overexpressing *PsMYB2* with the naked eye. Moreover, intense pigmentation was observed on the stamens of all transgenic tobacco flowers (lines 3, 5, and 6). The anthocyanin accumulation in the petal and stamen of lines 5 and 6 was much greater than that of the wild type. Although there was no difference in the anthocyanin content in the petals between line 3 transgenic tobacco and the wild type, the anthocyanin content in the stamen of the line 3 transgenic tobacco was significantly higher than that of the wild type, which was about 5 times that of the wild type.

As higher pigmentation in the petal and stamen of transgenic tobacco flowers, we extracted RNA of the transgenic and wild-type plants for further gene expression analysis. As shown in Figure 7C, except for line 3, the transcription levels of multiple anthocyanin biosynthetic genes were upregulated in flower petals. The expression levels of *NtCHS*, *NtF3H*, *NtANS*, and *NtUFGT* in line 5 were 3.30 times higher than those of the wild type. In stamens (Figure 7D), *NtCHS* and *NtANS* were upregulated in all transgenic tobacco lines. Particularly, *NtCHS* gene expression level in lines 3, 5, and 6 were 19.65, 24.08, and 12.10 times that of wild type, respectively. Furthermore, except for the petal of line 3, the two bHLH regulatory genes, *NtAn1a* and *NtAn1b*, involved in the regulation of tobacco anthocyanin production were all greatly upregulated in the *PsMYB2* transgenic petal and stamen (Figures 7C,D). Gene expression level of *NtAn1a* in stamens of lines 3, 5, and 6 was 23.83, 20.69, and 21.91 times higher than that of the wild type, respectively (Figure 7D).

### *PsMYB2* Induced Anthocyanin Accumulation of Transgenic *Arabidopsis* Seedlings in Response to Glucose Treatment

**FIGURE 8:**
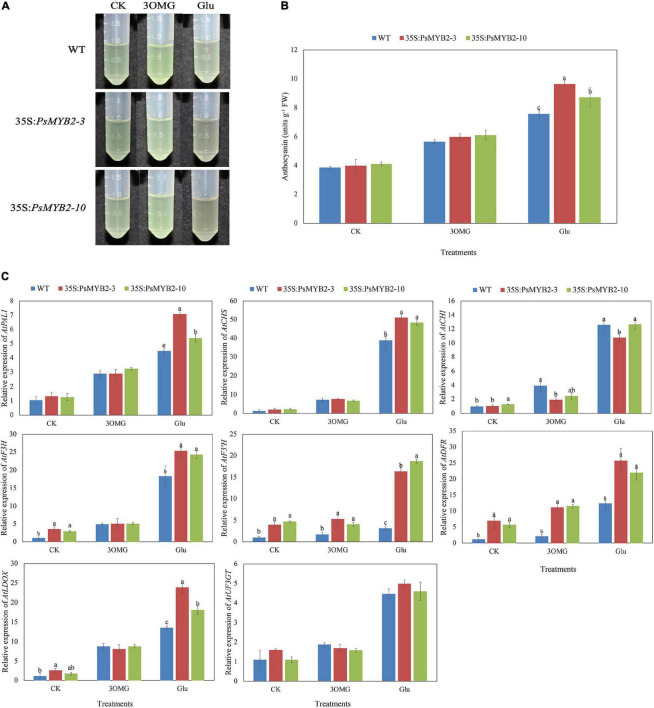
Overexpression of *PsMYB2* increased the ability of glucose-induced anthocyanin accumulation in *Arabidopsis*. **(A)** Anthocyanin extraction solutions for *Arabidopsis* seedlings under three treatments. **(B)** Total anthocyanin content in *Arabidopsis* seedlings under three treatments. **(C)** The relative expression levels of anthocyanin biosynthetic pathway genes in *Arabidopsis* under three treatments. Different small letters indicated significant differences among *PsMYB2* transgenic lines and wild-type plants according to Duncan’s multiple range test at *p* < 0.05. WT, wild-type plant; 35S:*PsMYB2* (3 and 10), plant overexpressing *PsMYB2*.

A total of two independent T3 homozygous of *PsMYB2* transgenic *Arabidopsis* were used to study the role of *PsMYB2* under glucose treatment in transgenic *Arabidopsis* seedlings. In the experiment, 3OMG was introduced as an osmotic control, and no glucose added was the control treatment (CK). As shown in Figure 8B, there were no differences in anthocyanin content between 35S:*PsMYB2* lines and wild-type seedlings in CK treatment. The anthocyanin accumulations were increased by 3OMG and glucose treatments, but the TA content under glucose treatment increased the most (Figure 8B). Under the osmotic control of 3OMG, the anthocyanin content of the 35S:*PsMYB2* lines 3 and 10 was 5.97 and 6.09 units g^–1^, respectively, which had no difference with the wild-type plants. Compared to the wild type, the TA content of 35S:*PsMYB2* transgenic lines 3 and 10 under glucose treatment was 9.66 and 8.71 units g^–1^, respectively, which were higher than that of wild-type seedlings (Figure 8B), and the anthocyanin extraction solutions of 35S:*PsMYB2* lines were much darker than the wild type (Figure 8A).

Comparison of 35S:*PsMYB2* lines with wild-type plants showed that the overexpression of *PsMYB2* enhanced the anthocyanin accumulation of seedlings treated with glucose. We further extracted RNA of samples under different treatments and detected the gene expression changes in 35S:*PsMYB2* lines and wild-type seedlings (Figure 8C). In the CK treatment with no sugar added, genes including *AtF3H*, *AtF3′H*, and *AtDFR* were highly expressed in the 35S:*PsMYB2* lines compared with the wild type. For 3OMG treatment, the expression levels of most structural genes in both transgenic and wild-type *Arabidopsis* were upregulated. However, the increase in expression levels of several genes in *PsMYB2* transgenic *Arabidopsis* was lower than that of wild type (Figure 8C). Under 3OMG treatment, only gene expression levels of *AtF3′H* and *AtDFR* in 35S:*PsMYB2* lines were higher than that of the wild type. Different with 3OMG treatment, although the expression levels of all structural genes in both wild type and 35S:*PsMYB2* lines were increased under glucose treatment, the genes including *AtPAL1*, *AtCHS*, *AtF3H*, *AtF3′H*, *AtDFR*, and *AtLDOX* in *PsMYB2* transgenic *Arabidopsis* were higher than those of the wild type. Particularly, the gene expression level of *AtF3′H* in lines 3 and 10 under glucose treatment was 5.25 and 6.02 times that of wild-type *Arabidopsis*, respectively (Figure 8C).

## Discussion

### Glucose Supply Promote Anthocyanin Accumulation in *P. suffruticosa* ‘Tai Yang’ Cut Flower

Cases of exogenous sugar supply enhancing the coloration of detached tissues or organs, including flowers, fruits, and roots, have been found in many plants, such as oriental lily (Han, 2003), phlox (Sankhla et al., 2005), strawberry (*Fragaria ananassa*) (Li et al., 2019), and radish (Hara et al., 2003). Similarly, in this study, glucose supply greatly enhanced the pigmentation of *P. suffruticosa* ‘Tai Yang’ cut flowers during postharvest development. At the best viewing period (S5), the flowers treated with glucose were more flamboyant, with uniform coloration of the middle petals (Figures 1B,C), matching with the color values of higher redness (a*), yellowness (b*), chroma (C*), and lower lightness (L*) (Figure 2). With the opening of cut flowers, the accumulations of anthocyanin and soluble sugar content in flower petals all increased (Figures 3A,B). Anthocyanin production occurring simultaneously with the accumulation of sugars was also found in radish hypocotyls (Hara et al., 2003), *Begonia semperflorens* (Zhang et al., 2013), apricot (*Prunus armeniaca*) (Huang et al., 2019), and grape berries (Yu et al., 2020). Sugars that have a special promotion effect on specific anthocyanin compositions are found in many studies. For example, during the storage, sucrose treatment specifically promoted pelargonidin derivatives synthesis in strawberry fruit (Li et al., 2019). In pigmented cells of *Vitis vinifera* suspension cultures, peonidin 3-glucoside was enhanced by higher sucrose levels (Bao Do and Cormier, 1991). Different from the previous studies, glucose had a wide range of promotion effects on anthocyanin compositions including Pg3G5G, Pg3G, and Pn3G5G in this study (Figures 3C–E).

Sugars not only serve as energy and structural materials in anthocyanin biosynthesis but also promote its accumulation through signal pathways (Rolland et al., 2002; Li et al., 2019), and glucose as a signal molecule to regulate anthocyanin production has been studied in many plants (Hara et al., 2003; Solfanelli et al., 2006; Zheng et al., 2009). The research showed that the phosphorylation process of HXK was considered an important sensing and transduction process of sugar regulation pathways involved in glucose signal (Jang et al., 1997). Thus, the glucose analog 3OMG, which can be transported inside the cell but not substrates for HXK (Colombo et al., 1978), is often introduced to distinguish glucose signal regulation. In this study, 3OMG treatment had a continuous promotion effect on anthocyanin accumulation from S2 to S5, including the compositions of Pg3G5G and Pn3G5G (Figures 3C,E), which was different from previous studies that 3OMG treatment hardly enhanced anthocyanin accumulation (Hara et al., 2003; Solfanelli et al., 2006; Zhang C. et al., 2015). The sugars in vase solution (metabolized sugar and non-metabolized sugar) could improve the water absorption of cut flowers and increase the osmotic concentration of petals, therefore maintaining the cut flowers at a good water balance condition (Ho and Nichols, 1977; Pun and Ichimura, 2003). 3OMG in this study might work as osmotic pressure and increase the water absorption of flower branches, so that the anthocyanin accumulations were improved. According to the research of Moalem-Beno et al. (1997), 3OMG also slightly promoted the anthocyanin content in the petunia corolla. Although 3OMG treatment had anthocyanin promotion effect on *P. suffruticosa* ‘Tai Yang’ cut flower, the color and anthocyanin content of flower petals under 3OMG treatment were lower than those of glucose (Figures 1B,C, 3), which suggested that glucose promoted anthocyanin production was not due to osmotic regulation.

Mannose is a kind of poorly metabolized glucose analog but can be phosphorylated by hexokinases to mannose-6P, modulating some sugar-regulatory processes (Loreti, 2001). Like the previous study of Zhang C. et al. (2015), the middle part of the petals showed an abnormal coloration under mannose treatment (Figure 1C), which might be related to the toxic effect of mannose or mannose-6P (Loreti, 2001). However, the TA content overall was increased by mannose treatment with higher accumulations of Pg3G5G and Pn3G5G (Figures 3B,C,E), which was different from the result that mannose hardly influenced the accumulation of anthocyanin in radish hypocotyls (Hara et al., 2003). In grape berry cells (Vitrac et al., 2000) and petunia flowers (Neta-Sharir et al., 2000), mannose could function like glucose and sucrose to regulate some sugar-regulatory processes at low concentrations and induce anthocyanin biosynthesis.

### Several Structural Genes Involved in Anthocyanin Biosynthesis Were Induced by Glucose Treatment in *P. suffruticosa* ‘Tai Yang’ Cut Flower During Postharvest Development

Studies showed that sugar-induced anthocyanin accumulation was through the upregulation of genes related to the anthocyanin biosynthetic pathway (Rolland et al., 2002; Van den Ende and El-Esawe, 2014; Abdullah et al., 2018). During the postharvest storage of strawberries, sucrose promoted anthocyanin production accompanied by higher CHS activity in fruit (Li et al., 2019). In broccoli sprouts, the gene expression levels of *CHI*, *F3H*, *DFR*, *LDOX*, and *GST* were promoted by sucrose treatment with the anthocyanin content increased (Guo et al., 2011). Similarly, several structural genes, including *PsF3H*, *PsF3′H*, *PsDFR*, *PsAOMT*, and *PsUF5GT*, were upregulated under glucose treatments (Figure 4), which was in accordance with the anthocyanin enhancement. In grape berry, the expression level of *F3H* was transiently induced from 2 to 4 h by different sugars, including glucose 100 mM, fructose 100 mM, and sucrose 150 mM (Zheng et al., 2009). During the treatment, *PsF3H* showed a continuous responding behavior to glucose treatment from S2 to S5 (Figure 4). It might be due to the stronger promotion effect of glucose in *P. suffruticosa*, like the previous study in Zhang C. et al. (2015). It is known that *PsF3′H* is a crucial gene for cyanidin-derived anthocyanin biosynthesis (Shi et al., 2015), and *PsAOMT* is responsible for converting cyanidin into peonidin (Du et al., 2015). Thus, these two genes are the nodes for the production of Pn3G5G and Pn3G in *P. suffruticosa* ‘Tai Yang.’ Under glucose treatment, *PsAOMT* kept higher expression levels from S3 to S5 and *PsF3′H* showed a much higher expression level at the S5 (Figure 4). We believed that the increased expression levels of *PsAOMT* and *PsF3′H* were directly related to the higher contents of Pn3G5G and Pn3G at the full opening stage in glucose-treated flowers (Figures 3E,F). Similarly, the previous study in *P. suffruticosa* ‘Luoyang Hong,’ a cyanidin-based purple-red color flower, found that the expression level of *PsF3′H* was increased by glucose treatment, showing a positive correlation with TA production (Zhang C. et al., 2015). *PsUF5GT* is a gene functioning in the biosynthesis of 3G5G-type anthocyanin in tree peony (Shu et al., 2008). We also observed the accordance between the expression level of *PsUF5GT* and the accumulations of Pg3G5G and Pn3G5G in glucose-treated flowers from S3 to S5 (Figures 3C,E, 4), suggesting that *PsUF5GT* could be a key gene in the glucose-induced anthocyanin accumulation in *P. suffruticosa* ‘Tai Yang.’ *UFGT* that played an essential role in the sugar-induced anthocyanin accumulation was also found in grape berries through genome-wide transcriptome analysis (Dai et al., 2013).

According to the previous study in petunia, 3OMG applied with GA3 slightly promoting *CHS* expression was considered to partly benefit from the modifications in the osmotic potential of the cells (Moalem-Beno et al., 1997). In this research, among the five structural genes upregulated by glucose treatments, we found that only *PsDFR* showed greater sensitivity to osmotic pressure (Figure 4). In contrast, the gene expression levels of *PsF3H*, *PsF3′H*, *PsAOMT*, and *PsUF5GT* under glucose treatment were much better than that of osmotic treatment overall (Figure 4), indicating that these genes were mainly regulated by glucose signal rather than osmotic control. *PsF3′H* was the only gene continuously promoted by mannose treatment from S2 to S5 (except for S4 with no significance) (Figure 4). Mannose, simulating sugars, enhanced the anthocyanin accumulation through the hexokinase-dependent (HXK-dependent) pathway with the upregulation of *F3H* expression in grape (Zheng et al., 2009). In this study, anthocyanin enhancement of Pn3G5G might be related to the upregulation of *PsF3′H* by mannose *via* the HXK-dependent pathway.

### Glucose Treatment Showed a Stronger Promotion Effect on Regulatory Genes and *PsMYB2* Might Be a Crucial Regulatory Gene in Response to Glucose Signal

Anthocyanin regulatory genes are the important mediators that lead to the initiation of anthocyanin biosynthesis in response to various developmental and environmental regulations (Dooner et al., 1991), as well as an important role in sugar-induced anthocyanin production (Teng et al., 2005; Dai et al., 2013; Ai et al., 2016). In *Arabidopsis*, sucrose specifically induced the anthocyanin biosynthesis, and the expression level of *AtMYB75* was upregulated by several 100-folds (Solfanelli et al., 2006). However, the *Arabidopsis*, whose *AtMYB75* protein function was almost lost due to mutation, hardly responded to sucrose with less anthocyanin accumulation in seedlings, suggesting an indispensable role of *AtMYB75* in sucrose-induced anthocyanin accumulation (Solfanelli et al., 2006). High glucose medium enhanced the anthocyanin level in grape berry, and genome-wide transcriptome analysis suggested that the regulatory genes played a curial role in this process (Dai et al., 2013). In our study, we believed that TFs including *PsMYB2*, *PsbHLH1*, *PsbHLH3*, *PsWD40-1*, and *PsWD40-2* were the important components in glucose-induced anthocyanin biosynthesis of *P. suffruticosa* ‘Tai Yang’ cut flowers since they kept higher expression levels throughout the flower opening process (S2–S5) (Figure 5). In addition, not all regulatory genes responded to glucose treatment. *PsMYB57*, which had been identified to be a positive regulator of anthocyanin biosynthesis in tree peony (Zhang et al., 2020), was highly expressed under 3OMG treatment rather than glucose treatment (Figure 5), indicating that *PsMYB57* might play important role in osmotic regulation. Besides, we suggested that *PsMYB114L* might play a weaker role in postharvest anthocyanin biosynthesis, since it was not highly expressed under glucose, 3OMG, or mannose treatments (Figure 5).

Among five TFs promoted by glucose, we found that only *PsMYB2* was highly expressed under mannose but less responsive to 3OMG treatment (Figure 5), which indicated that *PsMYB2* was likely to be an important transcription factor for glucose-induced anthocyanin biosynthesis through signal pathway. In the previous study of Zhang C. et al. (2015), the regulatory gene *PsMYB2* was also upregulated by glucose and mannose treatments but hardly promoted by 3OMG during the whole flower opening process. Therefore, we conducted further research on *PsMYB2*.

### *PsMYB2* Is a Positive Transcription Factor in Anthocyanin Biosynthesis

Flower color is an important economic trait for *P. suffruticosa*, and high color quality during the vase period tents to attract more people to purchase it. Studies show that R2R3-MYBs TFs play the essential roles in regulating anthocyanin biosynthesis (Koes et al., 2005). According to the gene expression analysis among four treatments, we suspected that *PsMYB2* might be a vital transcription factor that promoted the biosynthesis of anthocyanin in response to glucose signal. Since the function of *PsMYB2* had not been studied by others, we first examined its role in anthocyanin biosynthesis. Phylogenetic analyses indicated that the PsMYB2 protein was different from MYB TFs identified in tree peony recently, which participated in the regulation of anthocyanin biosynthesis, such as *PsMYB57* (Zhang et al., 2020), *PsMYB58* (Zhang et al., 2021), and *PsMYB114L* (Zhang et al., 2019). It belonged to a small cluster including *VvMYB5a/b*-like TFs (Figure 6B). Furthermore, sequence alignment found that *PsMYB2* not only had R2R3 MYB domain, bHLH interaction motif, and C1 motif (Figure 6A) but also contained a C3 motif that was specifically identified in the members of the *VvMYB5a/b* cluster (Yan et al., 2021), indicating a similar function that *PsMYB2* may have.

The overexpression of *PsMYB2* in tobacco showed similar phenotypic changes to the studies of *VvMYB5a/b*-like genes overexpressing in tobaccos. Anthocyanin accumulation in petal and stamen was enhanced in *PsMYB2* transgenic tobacco accompanied with higher anthocyanin content (Figures 7A,B), just like tobaccos overexpressing with *VvMYB5a* (Deluc et al., 2006), *VvMYB5b* (Deluc et al., 2006), *EsMYB9* (Huang et al., 2013), or *FhMYB5* (Li et al., 2018). Further gene expression analysis explained the increased anthocyanin accumulation in *PsMYB2* transgenic tobacco. In petals, most of the anthocyanin biosynthetic genes were upregulated (Figure 7C). Although genes upregulated in stamens were less, the expression levels of *NtCHS* and *NtANS* genes were strongly upregulated (Figure 7D). Similarly, almost all anthocyanin biosynthetic genes, including *NtCHS*, *NtCHI*, *NtF3H*, *NtDFR*, and *NtANS*, were remarkably upregulated in tobaccos overexpressing with *VvMYB5a* or *VvMYB5b* (Deluc et al., 2006, 2008). Besides, we found that the two bHLH regulatory genes, *NtAn1a* and *NtAn1b* that regulated the anthocyanin biosynthesis of tobacco, were also upregulated in the *PsMYB2* transgenic petals and stamens (Figures 7C,D). Similarly, the enhancement of *NtAn1a* and *NtAn1b* was also found in tobaccos overexpressing with *VvMYB5a/b*-like TFs including *EsMYB9*, *McMYB12*, and *LcMYB5* (Huang et al., 2017; Tian et al., 2017; Lai et al., 2019). Combined with the bHLH motif found in the *PsMYB2* protein domain (Figure 6A) and the research that MYB usually interacted with a bHLH protein forming a complex to regulate anthocyanin structural genes (Yan et al., 2021), we thought that *PsMYB2* might be able to interact with *NtAn1a* or *NtAn1b*, thus leading to the upregulation of *NtAn1a* and *NtAn1b* in tobacco. In addition, we noticed that the tobacco petals of transgenic line 3 did not appear darkening color, which may be related to the low expression levels of *NtAn1a* and *NtAn1b* in the petals of this line.

In conclusion, we suggested that *PsMYB2* was a positive transcription factor in anthocyanin biosynthesis, as well as a new member of the *VvMYB5b* cluster.

### Overexpression of *PsMYB2* Enhanced the Ability of Glucose-Induced Anthocyanin Accumulation in *Arabidopsis*

In *Arabidopsis*, gene changes leading to the altered responding ability to sugar also showed changed ability of sugar-induced anthocyanin production (Mita et al., 1997a,b; Baier et al., 2004). For example, *TTG1*, a gene encoding WD40 protein, was a key component for sucrose-induced anthocyanin production, and sucrose hardly induced anthocyanin accumulation in the *ttgl* mutant (Shirley et al., 1995). Sucrose-induced anthocyanin biosynthesis depended on the function of the AtMYB75 protein, and the loss of AtMYB75 activity caused a reduced response to sucrose-induced anthocyanin accumulation in *Arabidopsis* (Teng et al., 2005). Oppositely, in this study, overexpressing *PsMYB2* in *Arabidopsis* increased the sensitivity of seedlings to glucose treatment. Under glucose treatment, the anthocyanin content of *PsMYB2* transgenic *Arabidopsis* seedlings was higher than that of wild-type seedlings (Figure 8B), in accordance with much darker anthocyanin extraction solutions (Figure 8A). This result was similar to the previous research in petunia that transgenic lines overexpressing R2R3-MYB (*RsMYB1*) isolated from *Raphanus sativus* showed greater anthocyanin accumulation in response to sucrose treatment compared to wild-type plants (Ai et al., 2016). Further gene expression analysis showed that the expression levels of anthocyanin biosynthetic genes were upregulated under glucose treatment both in wild type and *PsMYB2* transgenic *Arabidopsis*. But the transcript of multiple structural genes including *AtPAL1*, *AtCHS*, *AtF3H*, *AtF3′H*, *AtDFR*, and *AtLDOX* in *PsMYB2* transgenic *Arabidopsis* was much higher than that of wild-type seedlings (Figure 8C). Similarly, the transcript levels of the anthocyanin biosynthetic genes, including *PAL*, *CHS*, *CHI*, *F3H*, *DFR*, and *ANS*, were elevated in *RsMYB1* transgenic plants under sucrose treatment (Ai et al., 2016). Besides, there were no differences in anthocyanin content between the *PsMYB2* transgenic and wild-type plants under the 3OMG treatment (osmotic control), illustrating that glucose-induced anthocyanin accumulation was not due to osmotic pressure. Overall, we suggested that the overexpression of *PsMYB2* increased the sensitivity of seedlings to glucose treatment leading to the higher anthocyanin accumulation in *Arabidopsis* seedlings.

## Conclusion

Exogenous glucose supply had a good color retention effect on *P. suffruticosa* ‘Tai Yang’ cut flower during postharvest development, and it was mainly through glucose signal pathway rather the osmotic pressure. Multiple anthocyanin biosynthetic genes were upregulated by glucose treatment, in accordance with anthocyanin accumulations of Pg3G, Pg3G5G, and Pn3G5G. *PsMYB2* was a new positive transcription factor for anthocyanin biosynthesis in tree peony, as well as a new member of the *VvMYB5b* cluster. More importantly, it could be a key component in glucose-induced anthocyanin accumulation through signal pathway in *P. suffruticosa* ‘Tai Yang’ cut flower.

## Data Availability Statement

The original contributions presented in this study are included in the article/Supplementary Material, further inquiries can be directed to the corresponding author.

## Author Contributions

LD and CZ designed and supervised this study. LZ and LY performed the experiments and analyzed the data. XK and YZ assisted with doing the experiments. LZ wrote the article. All authors read and approved the manuscript.

## Conflict of Interest

The authors declare that the research was conducted in the absence of any commercial or financial relationships that could be construed as a potential conflict of interest.

## Publisher’s Note

All claims expressed in this article are solely those of the authors and do not necessarily represent those of their affiliated organizations, or those of the publisher, the editors and the reviewers. Any product that may be evaluated in this article, or claim that may be made by its manufacturer, is not guaranteed or endorsed by the publisher.
