# Supplementary material for: Glucose Supply Induces PsMYB2-Mediated Anthocyanin Accumulation in Paeonia suffruticosa ‘Tai Yang’ Cut Flower
Source: Front Plant Sci. 2022 Jun 14;13:874526. doi: 10.3389/fpls.2022.874526 (PMC9237572; doi:10.3389/fpls.2022.874526)
Supplement: Supplementary file 1 [file Data_Sheet_1.docx]

***Supplementary Material***

**Supplementary Tables**

**Table S1.** Summary of primers used in this study.

| Primer name | Primer sequence (5’-3’) | Description |
| --- | --- | --- |
| qtPsCHI-F | AAATTCCCACCTGGTTCTTCTATTC | Primer used for qRT-PCR in tree peony |
| qtPsCHI-R | CTCCTTTGACCTTATCCATCCTTCA |  |
| qtPsCHS-F | AGCAGAGAACAACAAAGGGTCACG |  |
| qtPsCHS-R | TCAGCACCGACAATAACCGCAG |  |
| qtPsF3H-F | CCCAAGGTAGCCTACAACCAA |  |
| qtPsF3H-R | GAAAATCCCCCAGTCTTCACA |  |
| qtPsF3'H-F | AACTTGTTCACGGCAGGGACT |  |
| qtPsF3'H-R | GGCTTGGGCTAGGATTTTAGG |  |
| qtPsDFR-F | TGATAAAGCCAACAATAAATGGAATG |  |
| qtPsDFR-R | CACTCCAGCAGGTTTCGTCATACAC |  |
| qtPsANS-F | GCCCTCACTTTCATCCTCCACAAC |  |
| qtPsANS-R | AAAACTGCCCACGAAATCCTTACCT |  |
| qtPsAOMT-F | TAAGAAGGCTGGAGTGGAGCATAAG |  |
| qtPsAOMT-R | GGCATAGTTTTCCTTGTCAGCATCC |  |
| qtPsUF3GT-F | TGGGGTTGCCTTTTTATGGTCACTT |  |
| qtPsUF3GT-R | TCCACCTCCGATACTCTCTA |  |
| qtPsUF5GT-F | TCGTTTGGAAGCGTCTCTGTTTTAC |  |
| qtPsUF5GT-R | CCATTCCTTGCTTTTCCAAATCTTC |  |
| qtPsMYB2-F | GGTGGTCTTTGATTGCTGGGAG |  |
| qtPsMYB2-R | TCTGTTGGATTTGGGTTAGGGCTG |  |
| qtPsMYB57-F | TGGCGAAGGGGATCAAGTAG |  |
| qtPsMYB57-R | TTTGTCCTGTCTCTGGTGCA |  |
| qtPsMYB114L-F | GGGAGCGGGAATTCAGAGGTT |  |
| qtPsMYB114L-R | AGCCAAGAGTCCCCATCCAG |  |
| qtPsbHLH1-F | TAAAGCGAGCAATACACCAAATAAT |  |
| qtPsbHLH1-R | GGTGAACTTGGGATTCTCTCTAACT |  |
| qtPsbHLH3-F | GTCCATTTCAATTAGGCAACCAG |  |
| qtPsbHLH3-R | GAATACAAACGACATGCACACCA |  |
| qtPsWD40-1-F | GACATCCGCTTTCCGACGCT |  |
| qtPsWD40-1-R | AGTTGTTCAATCTCTGCCCCTGC |  |
| qtPsWD40-2-F | CCAACTCCGCTTCCTCACTTC |  |
| qtPsWD40-2-R | CTCAACCACACCCCTCTCCAC |  |
| qtPsubiquitin-F | GACCTATACCAAGCCGAAG |  |
| qtPsubiquitin-F | CGTTCCAGCACCACAATC |  |
| qtNtPAL-F | CGATAGACTTGAGGCATTTG | Primer used for qRT-PCR in tobacco |
| qtNtPAL-R | AGGTTCTCTTAGCGACTTG |  |
| qtNtCHS-F | AGCGAGCATAAGGTTGAG |  |
| qtNtCHS-R | ACCACCACTATGTCTTGTC |  |
| qtNtCHI-F | CTTTTCTCGCCGCTAAATG |  |
| qtNtCHI-R | TTTCTGCCACCTTCTCTG |  |
| qtNtF3H-F | GAGGCAATGGGCTTAGAG |  |
| qtNtF3H-R | TCAGTGTGTCGTTTCAGTC |  |
| qtNtF3'H-F | AGCCATAGTCAAGGAAACC |  |
| qtNtF3'H-R | CTCACAACTCTCGGATGC |  |
| qtNtDFR-F | TAAGAAGATGACAGGATGGATG |  |
| qtNtDFR-R | TGGCGGTATGATGCTAATG |  |
| qtNtANS-F | CTACATTCCAGCAACAAGTG |  |
| qtNtANS-R | GTCCCAGCCCAATAGAAAG |  |
| qtNtUFGT-F | GAGTGCATTGGATGCCTTTT |  |
| qtNtUFGT-R | CCAGCTCCATTAGGTCCTTG |  |
| qtNtAn1a-F | ACCATTCTCGAACACCGAAG |  |
| qtNtAn1a-R | TGCTAGGGCACAATGTGAAG |  |
| qtNtAn1b-F | CTTGAACACTTCTCAAACCGA |  |
| qtNtAn1b-R | TGCTAGGGCACAATGTGAAG |  |
| qtNtTub1-F | TCCGTGGTGATGTTGTG |  |
| qtNtTub1-R | TGGTGGCTGATAGTTGATAC |  |
| qtAtPAL1-F | CTTGGAACAGAGCTTTTGACCG | Primer used for qRT-PCR in Arabidopsis |
| qtAtPAL1-R | CGTGAAAACCTTGTCGAACTCTTC |  |
| qtAtCHI-F | CCGGTTCATCGATCCTCTTC |  |
| qtAtCHI-R | ATCCCGGTTTCAGGGATACTATC |  |
| qtAtCHS-F | GGCAAAGAAGCGGCAGTGAAG |  |
| qtAtCHS-R | CGGAAGGACGGAGACCAAGAAG |  |
| qtAtF3H-F | CAGATCGTTGAGGCTTGTGAGA |  |
| qtAtF3H-R | GACGAGTCATATCCGCCACTAAGT |  |
| qtAtF3'H-F | GCTCTCGCCGGAGTATTCAA |  |
| qtAtF3'H-R | CCAGCGACGCCTTGTAAATC |  |
| qtAtDFR-F | CTTTGTTCGTGCCACCGTTCG |  |
| qtAtDFR-R | TCCTTCCTCAGATAAATCAGCCTTCC |  |
| qtAtLDOX-F | GTTTGCAGCTTTTCTACGAGG |  |
| qtAtLDOX-R | TGAGCAAAAGTCCGTGGAGG |  |
| qtAtUF3GT-F | GTTAACGAACGGTTGTGGTTAG |  |
| qtAtUF3GT-R | GTGGAGATGTGTTTTGACTGAC |  |
| qtAtActin2-F | GCTGAGAGATTCAGATGCCCA |  |
| qtAtActin2-R | GTGGATTCCAGCAGCTTCCAT |  |
| PsMYB2-F | CATACAGGTAGAGTGAGAGAAAGGG | Primer used for sequence cloning |
| PsMYB2-R | CACAACAATACAACACGAAAGAGAA |  |
| GFPPsMYB2-F | ACGAACGATACTCGAGATGAGAAACCCTACATCTGGGTCTG | Primer used for vector construction |
| GFPPsMYB2-R | TCACTAGTACGTCGACGCAGAAATGCTTTTCAACATGCTC |  |
| 121-PsMYB2-F | GGACTCTAGAGGATCCATGAGAAACCCTACATCTGGGTCTG |  |
| 121-PsMYB2-R | GATCGGGGAAATTCGAGCTCTCAGAAATGCTTTTCAACATGCTC |  |

**Table S2.** Anthocyanin content of different compositions in *P. suffruticosa* ‘Tai Yang’ flowers at stage5 that grew naturally in the field condition

| Cultivar | Petal color | Anthocyanin content of different compositions (ug g^-1^) | | | | |
| --- | --- | --- | --- | --- | --- | --- |
|  |  | Pg3G5G | Pg3G | Pn3G5G | Pn3G | Total |
| *Paeonia suffruticosa* ‘Tai Yang’ | Red | 2132.93±46.73 | 779.21±23.56 | 152.71±2.49 | 31.7±0.4 | 3096.56±69.52 |

Pg3G5G, pelargonidin-3,5-di-O-glucoside; PG3G, pelargonidin 3-O-glucoside; Pn3G5G, peonidin-3,5-di-O-glucoside; Pn3G, peonidin-3-O-glucoside.


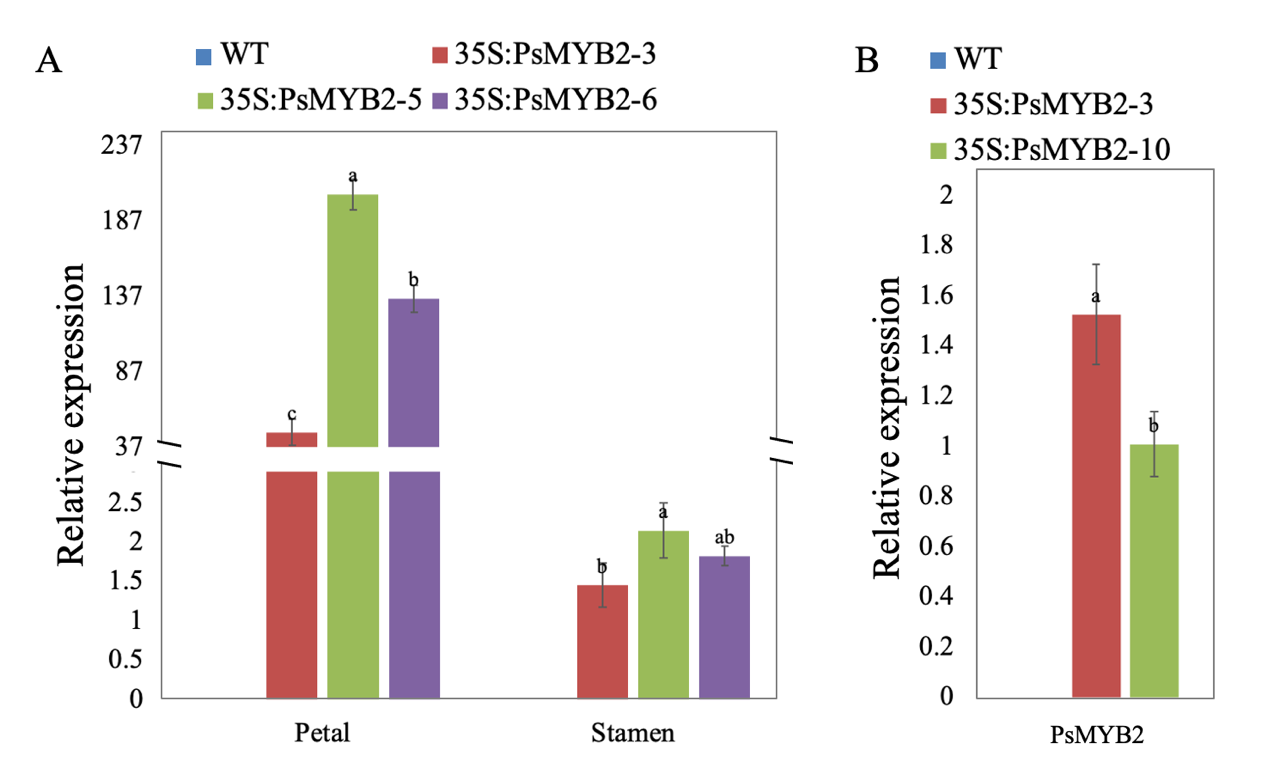


**Figure S1.** Expression levels of *PsMYB2* in tobacco (A) and *Arabidopsis* (B) indicated that *PsMYB2* was successfully overexpressed into tobacco and *Arabidopsis*.
